# Identification of Antibacterial Components and Modes in the Methanol-Phase Extract from a Herbal Plant *Potentilla kleiniana* Wight et Arn

**DOI:** 10.3390/foods12081640

**Published:** 2023-04-13

**Authors:** Yingping Tang, Pan Yu, Lanming Chen

**Affiliations:** 1 Key Laboratory of Quality and Safety Risk Assessment for Aquatic Products on Storage and Preservation (Shanghai), Ministry of Agriculture and Rural Affairs of the People’s Republic of China, Shanghai 201306, China; 2 College of Food Science and Technology, Shanghai Ocean University, Shanghai 201306, China

**Keywords:** *Potentilla kleiniana* Wight et Arn, antibacterial component, antibacterial mode, pathogenic bacteria, transcriptome, traditional Chinese herb

## Abstract

The increase in bacterial resistance and the decline in the effectiveness of antimicrobial agents are challenging issues for the control of infectious diseases. Traditional Chinese herbal plants are potential sources of new or alternative medicine. Here, we identified antimicrobial components and action modes of the methanol-phase extract from an edible herb *Potentilla kleiniana* Wight et Arn, which had a 68.18% inhibition rate against 22 species of common pathogenic bacteria. The extract was purified using preparative high-performance liquid chromatography (Prep-HPLC), and three separated fragments (Fragments 1–3) were obtained. Fragment 1 significantly elevated cell surface hydrophobicity and membrane permeability but reduced membrane fluidity, disrupting the cell integrity of the Gram-negative and Gram-positive pathogens tested (*p* < 0.05). Sixty-six compounds in Fragment 1 were identified using Ultra-HPLC and mass spectrometry (UHPLC-MS). The identified oxymorphone (6.29%) and rutin (6.29%) were predominant in Fragment 1. Multiple cellular metabolic pathways were altered by Fragment 1, such as the repressed ABC transporters, protein translation, and energy supply in two representative Gram-negative and Gram-positive strains (*p* < 0.05). Overall, this study demonstrates that Fragment 1 from *P. kleiniana* Wight et Arn is a promising candidate for antibacterial medicine and food preservatives.

## 1. Introduction

Infectious diseases caused by pathogenic bacteria continue to be a global concern for public health, causing millions of deaths worldwide per year [1]. Since the introduction of sulfonamides in 1933, a large number of antibiotics have been applied in clinics [2]. Nevertheless, in recent decades, the overuse and/or misuse of antibiotics have accelerated the spread of antibiotic-resistant bacteria, leading to ineffective drug treatment [3]. It was estimated that at least 700,000 people worldwide die each year due to antimicrobial resistance [4].

Pharmacophagous plants are recognized as a rich source of phytochemicals with antimicrobial potential [5]. Phytocompounds extracted from such plants are long known for their therapeutic uses, and characterized by safety and low toxicity [6]. The application of herbal products may be a better choice for the extensive and imprudent use of synthetic antibiotics [7]. For example, In China, approximately 34,984 native higher plant species have been recorded [8]. Of these, the herbal plant *Potentilla kleiniana* Wight et Arn was first recorded in the earliest pharmaceutical book “Divine Farmer’s Classic of Materia Medica” during the Warring States period (475–221 B.C.) in China. It belongs to the phylum of Angiospermae, the class of Dicotyledoneae, the order of Rosales Bercht. and J. Presl, and the family of Rosaceae Juss. *P. kleiniana* Wight et Arn is widely distributed in China, and many Asian countries such as Japan, India, Malaysia, Indonesia, and North Korea. Its whole plant has been used as a traditional Chinese medicine to treat fever, arthritis, malaria, insect and snake bites, hepatitis, and traumatic injury [9]. Recently, Zhou et al. identified bioactive components from *P. kleiniana* Wight et Arn with anti-human immunodeficiency virus-1 (HIV-1) protease activity [10]. Liu et al. developed an efficient method for the rapid screening and separation of α-glucosidase inhibitors from *P. kleiniana* Wight et Arn [11]. Li et al. [12] found antihyperglycemic and antioxidant effect of the total flavones of *P. kleiniana* Wight et Arn in streptozotocin induced diabetic rats, which may be helpful in the prevention of diabetic complications associated with oxidative stress [12]. However, to the best of our knowledge, there are few studies so far in the current literature on antibacterial activity of *P. kleiniana* Wight et Arn. Tao et al. [9] reported that total flavonoids from *P. kleiniana* Wight et Arn (TFP) inhibited biofilm formation and virulence factor production in methicillin-resistant *Staphylococcus aureus* (MRSA). The TFP also damaged cell membrane integrity of *Pseudomonas aeruginosa*. These results supported potential application of the TFP as a novel natural bioactive preservative in food processing [13]. Song et al. also reported that bioactive components extracted from *P. kleiniana* Wight et Arn showed antibacterial effects against *S. aureus*, *Candida albicans*, *P. aeruginosa*, and *Escherichia coli*, but not against the mold *Aspergillus niger* [14].

To further exploit bioactive nature products in *P. kleiniana* Wight et Arn, in the present study, we extracted bacteriostatic components in *P. kleiniana* Wight et Arn using the methanol and chloroform method [15,16]. Antimicrobial action modes of the methanol-phase extract were further investigated. The results of this study provide useful data for potential pharmaceutical application of *P. kleiniana* Wight et Arn against the common pathogenic bacteria.

## 2. Results and Discussion 

### 2.1. Antibacterial Activity of Crude Extracts from P. kleiniana Wight et Arn

Antibacterial substances in the fresh *P. kleiniana* Wight et Arn were extracted using the methanol and chloroform method [15,16]. The water loss rate of the fresh plant sample was 94.12% after freeze-drying treatment of the sample. The extraction rates of the methanol-phase and chloroform-phase crude extracts were 31.13% and 25.43%, respectively. As shown in Table 1, the chloroform-phase extract from *P. kleiniana* Wight et Arn had a 50.00% inhibition rate, which inhibited one species of Gram-positive bacterium *S. aureus*, and 10 species of Gram-negative bacteria, including *Bacillus cereus* A1-1, *B. cereus* A2-2, *Enterobacter cloacae* ATCC13047, *Salmonella typhimurium* ATCC15611, *Shigella dysenteriae* CMCC51252, *Shigella flexneri* CMCC51572, *Shigella sonnei* ATCC25931, *Vibrio cholerae* Q10-54, *Vibrio mimicus* bio-56759, *Vibrio parahemolyticus* ATCC33847, *V. parahemolyticus* B3-13, *V. parahemolyticus* B5-29, *V. parahemolyticus* B9-35, *V. parahemolyticus* A1-1, and *Vibrio vulnificus* ATCC27562 (Table 1).

**Figure 1:**
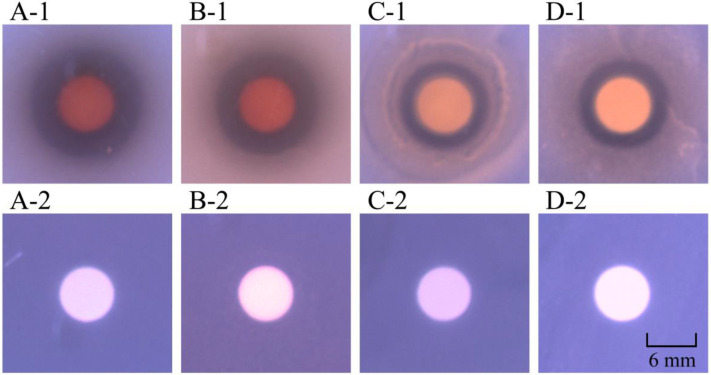
Inhibition activity of the methanol-phase crude extract from *P. kleiniana* Wight et Arn against the four representative bacterial strains. (**A-1**–**D-1**) *V. parahemolyticus* B5-29, *V. parahemolyticus* ATCC17802, *S. aureus* ATCC25923, and *S. aureus* ATCC8095, respectively. (**A-2**–**D-2**) corresponding negative controls, respectively.

Of note, the methanol-phase crude extract from *P. kleiniana* Wight et Arn inhibited the growth of 15 bacterial species, including one species of Gram-positive *S. aureus*, and 14 species of Gram-negative bacteria, *P. aeruginosa* ATCC9027, *S. typhimurium* ATCC15611, *S. dysenteriae* CMCC51252, *S. flexneri* CMCC51572, *S. flexneri* CMCC51574, *S. sonnei* ATCC25931, *V. alginolyticus* ATCC17749, *V. cholerae* Q10-54, *V. fluvialis* ATCC33809, *V. mimicus* bio-56759, *V. parahemolyticus* ATCC17802, and *V. vulnificus* ATCC27562, which showed a 68.18% inhibition rate (Table 1, Figure 1).

In this study, the methanol and chloroform extract method exhibited a broader antibacterial spectrum, consistent with our previous reports [15,16]. Previous studies also reported effective extraction of bioactive compounds from *P. kleiniana* Wight et Arn. For example, Tao et al. [13] extracted TFP in *P. kleiniana* Wight et Arn using an ethanol-water solution, and the obtained extract was further partitioned using petroleum ethers, chloroform and ethyl acetate. The extracted TFP inhibited survival and virulence of *P. aeruginosa*, and MRSA. Song et al. [14] extracted bioactive compounds from *P. kleiniana* Wight et Arn using ethanol and ethyl acetate, and the obtained extract showed antibacterial activity against *P. aeruginosa*, *S. aureus*, *C. albicans*, and *E. coli*. The difference in bioactive compounds extracted from *P. kleiniana* Wight et Arn using the different methods may explain the distinct antibacterial profiles between this study and the previous reports [13,14].

We further determined minimum inhibitory concentrations (MICs) of the crude extracts from *P. kleiniana* Wight et Arn, and the results are shown in Table 1. The MICs of the chloroform-phase extract ranged from 12.5 mg/mL to 50 mg/mL against the eleven species of the bacteria. Notably, for the methanol-phase extract, the MICs were between 1.56 mg/mL and 50 mg/mL against the fifteen bacterial species. Of these, the growth of *B. cereus* A2-2 and *V. parahemolyticus* ATCC17802 was the most strongly repressed by the methanol-phase extract with the MICs of 1.56 mg/mL, followed by *V. alginolyticus* ATCC17749, *V. mimicus* bio-56759, *V. parahemolyticus* B3-13, *V. parahemolyticus* B5-29, *V. parahemolyticus* B9-35, and *V. parahemolyticus* A1-1 with MICs of 3.13 mg/mL. In addition, the growth of *B. cereus* A1-1, *P. aeruginosa* ATCC9027, *S. typhimurium* ATCC15611, *S. flexneri* CMCC51572, *S. aureus* ATCC8095, and *V. parahemolyticus* B4-10 was also inhibited by the methanol-phase extract with lower MICs (6.25 mg/mL). Of these pathogens, for example, *V. alginolyticus* is a foodborne marine *Vibrio* that can cause gastroenteritis, otitis media, otitis externa, and septicemia in humans [17]. *V. mimicus* can also cause gastroenteritis in humans due to contaminated fish consumption and seafood [18]. *P. aeruginosa* is an opportunistic pathogen and can cause serious infections, especially in patients with compromised immune systems [19].

Recently, Song et al. [14] reported that the ethyl acetate extract of *P. kleiniana* Wight et Arn inhibited *E. coli*, *P. aeruginosa*, and *C. albicans*, with MICs of 5 mg/mL, 2.5 mg/mL, and 5 mg/mL, respectively. Tao et al. reported the MIC value of the TFP against MRSA was 20 μg/mL [9].

These results indicated that the methanol-phase crude extract had a higher inhibition rate (68.18%), showing a more broad inhibitory profile with much lower MICs (1.56–50 mg/mL) against the pathogens tested, as compared to the chloroform-phase crude extract (50.00%; 12.5–50 mg/mL). Thus, the methanol-phase crude extract was chosen for further analysis in this study.

### 2.2. Purification of the Methanol-Phase Crude Extract from P. kleiniana Wight et Arn

Based on the obtained results, a large amount of the methanol-phase crude from *P. kleiniana* Wight et Arn was prepared and further purified using Prep-HPLC analysis. As shown in Figure S1, three separated fragments (designated Fragments 1–3) were observed via scanning at OD_211_ for 12 min, including Fragment 1 (2.45 min), Fragment 2 (6.75 min), and Fragment 3 (9.83 min). The main peak of the methanol-phase crude was observed to occur at 2.45 min, wherein the absorption peak of Fragment 1 reached its maximum.

The three single fragments were subjected for antibacterial activity analysis. Fragment 1 had strong inhibitory effects on *V. parahemolyticus* ATCC17802, *V. parahemolyticus* B5-29, *V. parahemolyticus* B9-35, *V. parahemolyticus* B3-13, and *V. parahemolyticus* B4-10. In addition, the growth of the other six strains was also effectively repressed, including *B. cereus* A2-2, *V. parahemolyticus* A1-1, *S. flexneri* CMCC51572, *S. aureus* ATCC25923, *S. aureus* ATCC8095, and *S. aureus* ATCC6538 (Table 2). Of these, *V. parahaemolyticus* is a Gram-negative halophilic bacterium that can cause diseases in marine animals, leading to huge economic losses to the aquaculture. *V. parahaemolyticus* can also cause gastrointestinal infections and other health complications in humans [20]. *B. cereus* is a Gram-positive foodborne pathogen that can cause diarrhea and emesis [21]. *S. flexneri* is a Gram-negative intracellular pathogen that invades colonic cells and causes bloody diarrhea in humans [22]. *S. aureus* is a Gram-positive opportunistic pathogen leading to food poisoning as well as human and animal infectious diseases [23,24].

We also determined MICs of Fragment 1 against the four species of pathogenic bacteria (Table 2). The synergistic effect may explain the observed MICs of Fragment 1 (6.25–50 mg/mL), as compared to the methanol-phase extract from *P. kleiniana* Wight et Arn. Among the Gram-negative pathogens, *V. parahemolyticus* ATCC17802 and *V. parahemolyticus* B5-29 were the most sensitive strains to Fragment 1, with MICs of 6.25 mg/mL. For the Gram-positive pathogen, the growth of *S. aureus* ATCC8095 and *S. aureus* ATCC25923 was also effectively repressed, with MICs of 6.25 mg/mL and 12.5 mg/mL, respectively. 

Conversely, the other two peaks (Fragments 2 and 3) showed weak or no antibacterial activity. To further investigate possible antibacterial modes of Fragment 1, the two Gram-negative strains *V. parahemolyticus* ATCC17802 and *V. parahemolyticus* B5-29, and two Gram-positive stains *S. aureus* ATCC8095 and *S. aureus* ATCC25923 were chosen for the further analysis in this study.

### 2.3. Bacterial Cell Surface Hydrophobicity, Membrane Fluidity and Permeability Changes Triggered by Fragment 1 from P. kleiniana Wight et Arn

#### 2.3.1. Cell Surface Hydrophobicity

**Figure 2:**
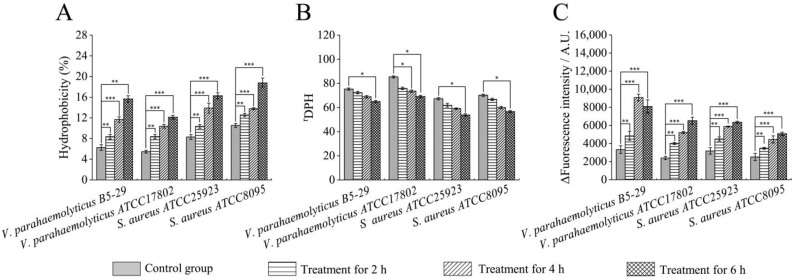
Effects of Fragment 1 (1× MIC) from *P. kleiniana* Wight et Arn on cell surface hydrophobicity, membrane fluidity and outer membrane permeability of the four bacterial strains. (**A**–**C**) cell surface hydrophobicity, membrane fluidity, and outer membrane permeability, respectively. *: *p* < 0.05; **: *p* < 0.01; and ***: *p* < 0.001.

Cell surface hydrophobicity is an important cellular biophysical parameter that affects cell surface interactions and cell–cell communication [25]. In this study, the hexadecane was used as a probe to assess cell surface hydrophobicity change. The difference between before and after the absorbance value of bacterial fluid can indicate the change of hydrophobicity, and the larger the difference, the more hydrophobicity of the surface [26]. The cell surface hydrophobicity of the four experimental groups (1× MIC of Fragment 1) was significantly increased, as compared to the control groups (*p* < 0.05) (Figure 2A). For instance, after being treated with Fragment 1 for 2 h, bacterial cell surface hydrophobicity was significantly increased, including *V. parahaemolyticus* B5-29 (8.62%, 1.42-fold), *V. parahaemolyticus* ATCC17802 (8.27%, 1.50-fold), *S. aureus* ATCC25923 (10.34%, 1.24-fold), and *S. aureus* ATCC8095 (12.20%, 1.19-fold) (*p* < 0.05). Increasing treatment time, the cell surface hydrophobicity was further increased. After the 4 h treatment, the cell surface hydrophobicity was the most significantly increased (11.97%, 1.97-fold) in the *V. parahaemolyticus* B5-29 treatment group. The highest increase (15.96%, 2.63-fold) was also observed in *V. parahaemolyticus* B5-29, after treatment for 6 h. The results indicated that Fragment 1 from *P. kleiniana* Wight et Arn can significantly increase the cell surface hydrophobicity of both Gram-negative *V. parahemolyticus* and Gram-positive *S. aureus* pathogens.

#### 2.3.2. Cell Membrane Fluidity

Cell membrane is a natural barrier to prevent extracellular substances from freely entering the cell [27]. In this study, as shown in Figure 2B, when compared to the control groups, the membrane fluidity of *V. parahaemolyticus* B5-29, *S. aureus* ATCC25923, and *S. aureus* ATCC8095 did not change significantly after treatment with Fragment 1 (1× MIC) for 2 h and 4 h. However, a significant decrease (1.16-fold, 1.25-fold, and 1.24-fold) was observed in these three treatment groups after treatment for 6 h, respectively (*p <* 0.05). In addition, a significant decrease in cell membrane fluidity was only found in *V. parahaemolyticus* ATCC17802 after treatment for 4 h (1.16-fold) and 6 h (1.24-fold), respectively (*p <* 0.05). These results indicated that Fragment 1 from *P. kleiniana* Wight et Arn can significantly reduce the cell membrane fluidity of both Gram-negative *V. parahemolyticus* and Gram-positive *S. aureus* pathogens.

#### 2.3.3. Cell Membrane Permeability

**Figure 3:**
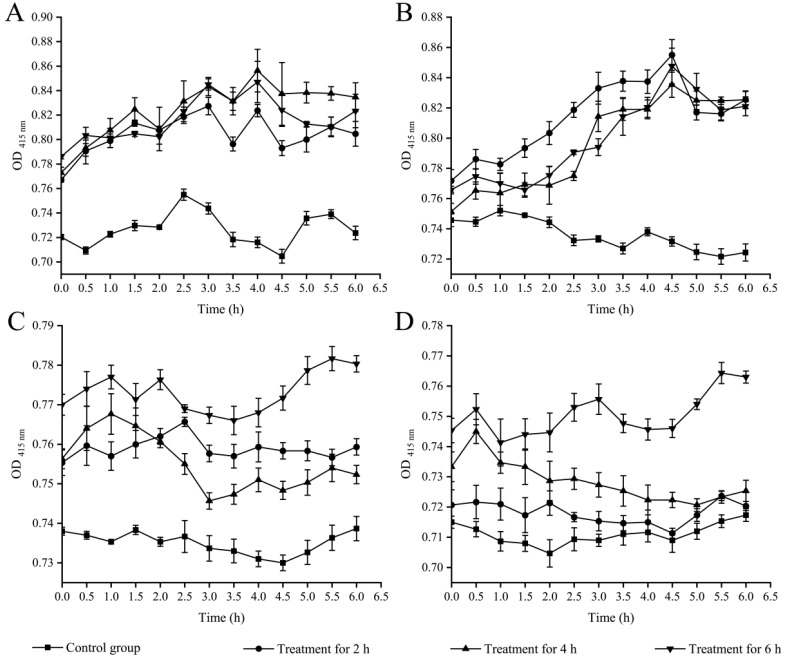
Effects of Fragment 1 (1× MIC) from *P. kleiniana* Wight et Arn on the bacterial inner cell membrane permeability. (**A**–**D**) *V. parahaemolyticus* B5-29, *V. parahaemolyticus* ATCC17802, *S. aureus* ATCC25923, and *S. aureus* ATCC8095, respectively. The treatment groups were overall significantly different from the control groups (*p* < 0.05), except the *S. aureus* ATCC8095 group treated for 2 h (**D**).

β-galactosidase is a macromolecular protein naturally found in the interior of cells that can hydrolyze the substrate o-nitrophenyl-β-D-galactopyranosi (ONPG) to galactose and o-nitrophenol in yellow. If the inner membrane of bacterial cells is damaged, ONPG will quickly enter the cell [28]. In this study, the ONPG was used as a probe to assess whether the bacterial inner membrane is damaged. As illustrated in Figure 3D, the inner cell membrane permeability of *S. aureus* ATCC8095 did not change significantly after treatment with Fragment 1 (1× MIC) from *P. kleiniana* Wight et Arn for 2 h (*p* > 0.05); conversely, significant increases were observed in *V. parahaemolyticus* B5-29, *V. parahaemolyticus* ATCC17802, and *S. aureus* ATCC25923 treatment groups (1.15-fold, 1.18-fold, and 1.04-fold), respectively (*p* < 0.05). After being treated for 4 h, the highest increase was found in *V. parahaemolyticus* B5-29 (1.22-fold). After treatment for 6 h, significant increases were also observed in *V. parahaemolyticus* B5-29, *V. parahaemolyticus* ATCC17802, *S. aureus* ATCC25923, and *S. aureus* ATCC8095 (1.20-fold, 1.17-fold, 1.07-fold, and 1.08-fold), respectively (*p <* 0.05). These results indicated that Fragment 1 from *P. kleiniana* Wight et Arn can significantly increase the inner cell membrane permeability of both Gram-negative *V. parahemolyticus* and Gram-positive *S. aureus* pathogens.

Outer membrane permeability was assessed by measuring the uptake of a hydrophobic fluorescent probe N-phenyl-1-naphthylamine (NPN) [29]. The outer membrane permeability increased significantly in the four treatment groups, after being treated with Fragment 1 for 2 h (1.38-fold to 1.66-fold) (*p* < 0.01), and 4 h (1.77-fold to 2.72-fold), respectively (*p* < 0.001) (Figure 2C). The highest increase was found in *V. parahaemolyticus* ATCC17802 (2.70-fold), after treatment for 6 h. These results indicated that Fragment 1 from *P. kleiniana* Wight et Arn can significantly increase the outer cell membrane permeability of the Gram-negative *V. parahemolyticus* and Gram-positive *S. aureus* pathogens. Recently, Tao et al. also reported that the TFP from *P. kleiniana* Wight et Arn increased cell membrane permeability of MRSA [13].

Taken together, the results of this study demonstrated that Fragment 1 (1× MIC) from *P. kleiniana* Wight et Arn can significantly increase the cell surface hydrophobicity and membrane permeability, but decreases the cell membrane fluidity of both Gram-negative *V. parahemolyticus* and Gram-positive *S. aureus* pathogens. Antibacterial compounds (e.g., flavonoids) in Fragment 1 from *P. kleiniana* Wight et Arn may have interacted with lipid components of the bacterial cell membrane. The disorder in lipid chains resulted in changed permeability and fluidity of the bacterial cell membrane [30]. The compounds may also have interacted with the bacterial cell surface proteins, leading to the altered nanomechanical properties, which consequently changed cell surface hydrophobicity and fluidity [31]. The two common pathogens *V. parahemolyticus* and *S. aureus* were chosen for further analysis in this study. The former is the leading sea foodborne pathogen worldwide [20], while the latter leads to food poisoning, as well as human and animal infections [23].

### 2.4. Bacterial Cell Surface Structure Changes Triggered by Fragment 1 from P. kleiniana Wight et Arn

**Figure 4:**
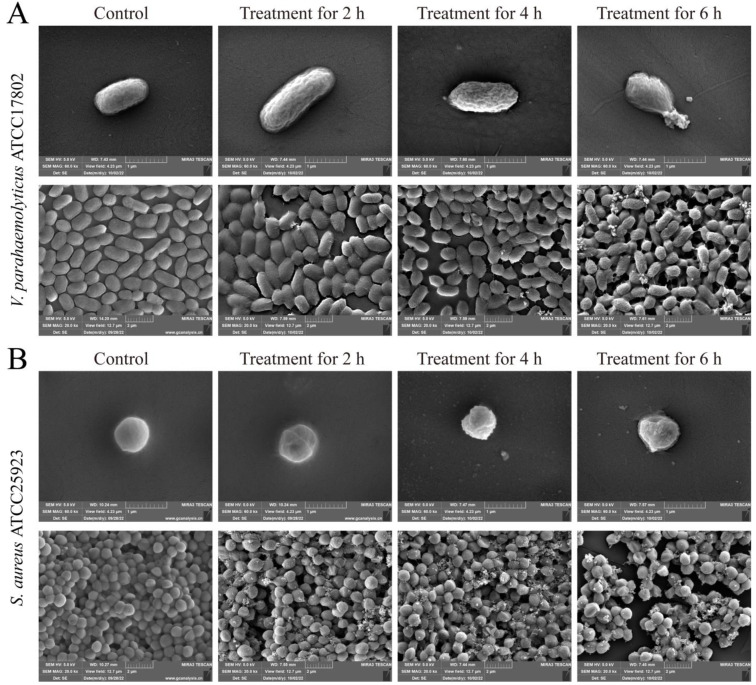
The SEM observation of cell surface structure of the two bacterial strains treated with the 1× MIC of Fragment 1 for different times. (**A**): *V. parahaemolyticus* ATCC17802; (**B**): *S. aureus* ATCC 25923.

Based on the obtained results in this study, the representative Gram-negative *V. parahaemolyticus* ATCC17802 and Gram-positive *S. aureus* ATCC25923 strains were chosen for further scanning electron microscope (SEM) analysis. As shown in Figure 4, the cells of *V. parahaemolyticus* ATCC17802 were intact in shape with a flat surface, showing a typical rod-like structure, while those of *S. aureus* ATCC25923 were also intact and clear, showing a typical spherical structure. In remarkable contrast to the control groups, the bacterial morphological structures were altered to varying degrees in the treatment groups triggered by Fragment 1 (1× MIC) for different times.

For the Gram-negative *V. parahaemolyticus* ATCC17802, its cell surface was slightly shrunken after being treated with Fragment 1 for 2 h. After 4 h of treatment, the cell surface was more wrinkled and was slightly depressed, the cell membrane was folded and some contents were exuded. After 6 h of the treatment, the cells were severely deformed and crumpled, with a large amount of content leaked.

For the Gram-positive *S. aureus* ATCC25923, its cell surface was rough and slightly wrinkled, but certain cells were depressed, with a small amount of content leaked after the treatment for 2 h. Upon the increased treatment time (4 h), more cells were obviously wrinkled and deformed with the irregularly spherical, and more content leaked out. The cell morphological structure was seriously damaged after being treated for 6 h.

These results demonstrated that Fragment 1 (1× MIC) from *P. kleiniana* Wight et Arn can severely damage the cell surface structure of both Gram-negative *V. parahaemolyticus* and Gram-positive *S. aureus* after treatment for 6 h.

### 2.5. Identification of Potential Antibacterial Compounds in Fragment 1 from P. kleiniana Wight et Arn

Potential antibacterial components in Fragment 1 from *P. kleiniana* Wight et Arn were further identified using UHPLC-MS analysis. As shown in Table 3, a total of 66 different compounds were identified. The highest relative percentage of the compounds was D-maltose (6.77%), followed by oxymorphone (6.29%), rutin (6.29%), D-proline (5.41%), and L-proline (5.41%). In addition, alkaloids, flavonoids, phenols, sesquiterpenoids, fatty acyls, and organic acids were also detected (Table 3).

Highly concentrated sugar solutions, such as the D-maltose identified in this study, are known to be effective antimicrobial agents [32]. Previous research has indicated that the antibacterial activity of phenanthrenes and derivatives, such as the oxymorphone identified in this study, was primarily related to the destruction of the bacterial cell wall structure [33]. Plant extracts contain a large number of bioactive compounds, mainly polyphenols including flavonoids and phenolic compounds. Flavonoids, such as the rutin identified in this study, could exert antibacterial activity via damaging the cytoplasmic membrane, inhibiting energy metabolism and synthesis of nucleic acids [34]. Tao et al. also reported the major compounds of the TFP were 3-O-methylducheside A, naringenin, rutin and quercetin [9,13]. Phenols, such as the p-octopamine identified in this study, are potent antibacterial agents against both Gram-positive and Gram-negative bacteria via the disruption of the bacterial membrane, leading to bacterial lysis and leakage of intracellular contents [35]. Indole alkaloids, such as the indole identified in this study, possess not only intriguing structural features but also biological/pharmacological activities e.g., antimicrobial activity [36]. Additionally, amino acids and its derivatives, such as the D-proline, L-proline, glutamic acid, 5-aminovaleric acid, lysine, pipecolic acid, and L-valine identified in this study, are a kind of antibacterial agent with the advantages of being not easily drug-resistant, and having low toxicity or harmless metabolites [37].

### 2.6. Differential Transcriptomes Triggered by Fragment 1 from P. kleiniana Wight et Arn

To obtain the genome-wide gene expression changes triggered by Fragment 1 from *P. kleiniana* Wight et Arn, we determined transcriptomes of the Gram-negative *V. parahaemolyticus* ATCC17802 and the Gram-positive *S. aureus* ATCC25923 pathogens treated with Fragment 1 (1× MIC) for 6 h using the Illumina RNA sequencing technology. A complete list of differently expressed genes (DEGs) in the two strains are available in the National Center for Biotechnology Information (NCBI) SRA database under the accession number PRJNA906658.

#### 2.6.1. The Major Changed Metabolic Pathways in *V. parahaemolyticus* ATCC17802

**Figure 5:**
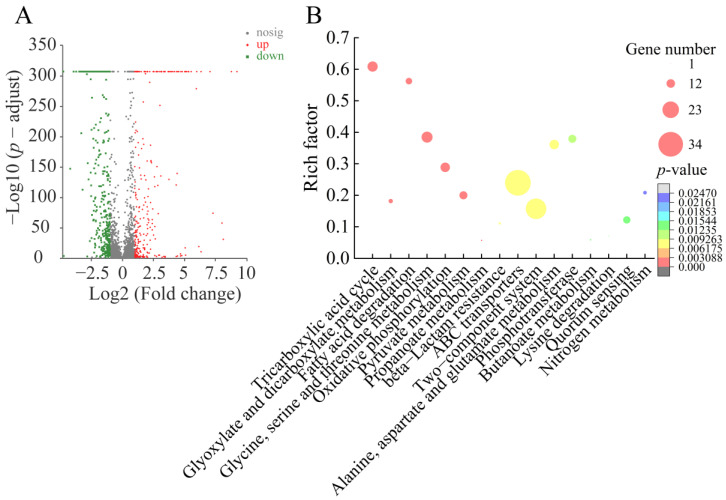
The major changed metabolic pathways in *V. parahaemolyticus* ATCC 17802 mediated by Fragment 1 from *P. kleiniana* Wight et Arn. (**A**) The Volcano plot of the DEGs. (**B**) The significantly altered metabolic pathways in the bacterium. Different colors represented significant levels of the enriched genes.

Approximately 13.07% (580 of 4436 genes) of *V. parahaemolyticus* ATCC17802 genes were differentially expressed in the treatment group, as compared to the control group. Of these, 238 DEGs showed higher transcriptional levels (fold change ≥ 2.0), whereas 342 DEGs were significantly down-regulated (fold change ≤ 0.5) (*p* < 0.05). Sixteen significantly altered metabolic pathways were identified in *V. parahaemolyticus* ATCC 17802, including the citrate cycle; glyoxylate and dicarboxylate metabolism; fatty acid degradation; glycine, serine, and threonine metabolism; oxidative phosphorylation; pyruvate metabolism; propanoate metabolism; beta-Lactam resistance; ABC transporters; two-component system; alanine, aspartate, and glutamate metabolism; phosphotransferase system (PTS); butanoate metabolism; lysine degradation; quorum sensing (QS); and nitrogen metabolism (Figure 5, Table 4).

In the citrate cycle, all the DEGs (*n* = 14) were significantly repressed (0.146-fold to 0.35-fold) (*p* < 0.05) in *V. parahaemolyticus* ATCC17802 after treatment by Fragment 1 from *P. kleiniana* Wight et Arn. For instance, the DEGs (*sucABCD, WU75_19785* and *WU75_19790*, *WU75_19795,* and *WU75_19800*), encoding a 2-oxoglutarate dehydrogenase, a dihydrolipoamide succinyltransferase, and succinyl-CoA synthetase subunits alpha and beta, respectively, were highly inhibited (0.146-fold, 0.133-fold, 0.134-fold, and 0.16-fold) (*p* < 0.05). Moreover, the DEGs (*sdhABCD, WU75_19775*, *WU75_19780*, *WU75_19765,* and *WU75_19770*) encoding a succinate dehydrogenase were also highly repressed (0.144-fold to 0.199-fold) (*p* < 0.05), which links two essential energy-producing processes, the citrate cycle and oxidative phosphorylation [38]. The inhibited key enzymes in the citrate cycle highlighted inactive energy production in *V. parahaemolyticus* ATCC17802 triggered by Fragment 1.

In the propanoate metabolism, all the DEGs (*n* = 2) were significantly inhibited (0.402-fold to 0.435-fold) in the *V. parahaemolyticus* ATCC17802 treatment group (*p* < 0.05). For example, the DEG (*prpC*, *WU75_15770*) encoding a 2-methylcitrate synthase was significantly inhibited (0.435-fold) (*p* < 0.05). It has been reported that the strategic inhibition of organic acid catabolism in *P. aeruginosa* through inhibition of PrpC activity may be a potent mechanism to halt the growth of this pathogen [39].

In the glyoxylate and dicarboxylate metabolism, five of the six DEGs were significantly repressed (0.129-fold to 0.277-fold) (*p* < 0.05). For instance, the DEGs (*aceAB*, *WU75_19150*, *WU75_19145*, and *WU75_00290*), encoding an isocitrate lyase and a malate synthase of the glyoxylate shunt (GS) carbon cycle, were significantly inhibited (0.315-fold to 0.370-fold) (*p* < 0.05). The GS could avoid unnecessary reactive oxygen species (ROS) generation by bypassing nicotinamide adenine dinucleotide (NADH) production, and respiration, eventually helping cells to survive in harsh conditions [40,41].

In the glycine, serine, and threonine metabolism, all the DEGs (*n* = 15) were significantly inhibited (0.113-fold to 0.495-fold) in *V. parahaemolyticus* ATCC17802 (*p* < 0.05). For example, the DEGs (*ectBAC*, *WU75_16140*, *WU75_16145*, and *WU75_16135*), encoding a diaminobutyrate-2-oxoglutarate aminotransferase, a 2% 2C4-diaminobutyric acid acetyltransferase, and an ectoine synthase, which are involved in the synthesis of ectoine that is commonly found in halophilic and halotolerant microorganisms to maintain cell osmotic balance [42]. Additionally, in the alanine, aspartate, and glutamate metabolism, ten of the thirteen DEGs were significantly down-regulated (0.037-fold to 0.466-fold) in *V. parahaemolyticus* ATCC17802 as well (*p* < 0.05). Conversely, the DEGs (*ansAB*, *WU75_20915*, and *WU75_01110*) were up-regulated (2.141-fold and 2.718-fold) (*p* < 0.05), which encoded a cytoplasmic asparaginase I and a L-asparaginase II. The asparaginase I is required for bacterial growth on asparagine as the sole nitrogen source [43], while asparaginases are important in maintaining nitrogen balance and the levels of amino acids within cells [43]. These results indicated that the amino acid synthesis was inhibited in *V. parahaemolyticus* ATCC17802 mediated by Fragment 1.

For the ABC transporters, 29 of the 35 DEGs were significantly down-regulated (0.106-fold to 0.491-fold) in *V. parahaemolyticus* ATCC17802 (*p* < 0.05). Of these, the DEGs (*proVXW*, *WU75_10380*, *WU75_10390*, and *WU75_10385*), encoding a choline ABC transporter ATP-binding protein, a choline ABC transporter substrate-binding protein, and a choline ABC transporter permease subunit that are responsible for the choline transport, were all significantly repressed (0.106-fold to 0.138-fold). The DEGs (*oppABCDF*, *WU75_12765*, *WU75_12770*, *WU75_12775*, *WU75_12780*, and *WU75_12785*) encoding a peptide ABC transporter substrate-binding protein, an oligopeptide transporter permease, a peptide ABC transporter permease, an oligopeptide transporter ATP-binding component, and a peptide ABC transporter ATP-binding protein, respectively, were all highly repressed (0.172-fold and 0.214-fold). Additionally, the DEGs (*yejABE*, *WU75_13090*, *WU75_07210*, *WU75_07220*, and *WU75_07215*) encoding a diguanylate cyclase, an ABC transporter permease subunit, and a peptide ABC transporter permease, respectively, were highly repressed as well (0.151-fold and 0.220-fold). The ABC transporter YejABEF is required for resistance to antimicrobial peptides and virulence of *Brucella melitensis* [44]. These results indicated that the inhibited ABC transporters likely led to the repressed substance transport and harmful substances discharged in *V. parahaemolyticus* ATCC17802.

In the oxidative phosphorylation, nine of the thirteen DEGs were significantly down-regulated in *V. parahaemolyticus* ATCC17802 (0.195-fold to 0.478-fold) (*p* < 0.05). Oxidative phosphorylation is a major metabolic pathway to obtain energy required for cell growth and proliferation [45] (Huang et al., 2019). For instance, the DEGs (*ccoNOQ*, *WU75_14575*, *WU75_14570*, and *WU75_14565*) were significantly inhibited (0.228-fold to 0.475-fold) (*p* < 0.05), which regulated the bacterial adhesion in environmental stresses in *V. alginolyticus* [45].

In the QS, most DEGs (*n* = 9) were significantly inhibited (0.109-fold to 0.484-fold) (*p* < 0.05), e.g., cytochrome c (*WU75_06010*), cytochrome B (*WU75_06015*), and peptidase S41 (*WU75_14570*). For instance, the cytochrome c mediates electron-transfer in the respiratory chain and acts as a detoxifying agent to dispose of reactive oxygen species (ROS) [46].

In contrast, in the PTS, nine of the eleven DEGs were significantly up-regulated (2.36-fold to 6.946-fold) in the *V. parahaemolyticus* ATCC17802 treatment group (*p* < 0.05). Of these, the DEGs (*fruA, WU75_14960*; *ulaA, WU75_00450*), encoding a PTS fructose transporter subunit IIBC and a PTS beta-glucoside transporter subunit IIBC, respectively, were highly up-regulated (5.096-fold and 6.946-fold) (*p* < 0.05).

In the nitrogen metabolism, most of the DEGs (*n* = 4) were significantly up-regulated (2.286-fold to 63.107-fold) (*p* < 0.05). Remarkably, the DEG (*hcp*, *WU75_08850*) encoding a hydroxylamine reductase was strongly up-regulated (63.107-fold) (*p* < 0.05), and is involved in the processes of scavenging hydroxylamine with NO detoxification [47].

In the two-component system, 19 DEGs were significantly inhibited (0.186-fold to 0.491-fold), whereas 9 DEGs were significantly enhanced (2.068-fold to 26.5-fold) (*p* < 0.05). The two-component system is one of the primary pathways by which bacteria adapt to environmental stresses [48]. For instance, the DEGs (*cpxAR, WU75_18570,* and *WU75_18575*) encoding a two-component sensor protein and a transcriptional regulator were strongly up-regulated (10.981-fold and 26.500-fold) (*p* < 0.05). The CpxAR is a key modulator of capsule export that facilitates *Actinobacillus pleuropneumoniae* survival in the host [49]. It also regulates cell membrane permeability and efflux pump activity and induces multidrug resistance (MDR) in *Salmonella enteritidis* [50].

Additionally, in the beta-lactam resistance, all the DEGs (*acrAB*, *WU75_09925*, *WU75_09315*, and *WU75_09310*) were strongly up-regulated (6.699-fold to 40.366-fold) in the *V. parahaemolyticus* ATCC17802 treatment group (*p* < 0.05), which encoded a multidrug efflux resistance nodulation division (RND) transporter periplasmic adaptor subunit and a multidrug transporter. The RND family efflux pumps, including the major pump AcrAB-TolC, are important mediators of intrinsic and evolved antibiotic resistance [51].

Taken together, these results indicated that Fragment 1 from *P. kleiniana* Wight et Arn can significantly change sixteen metabolic pathways in the Gram-negative *V. parahaemolyticus* ATCC17802, which consequently led to repressed substance transporting, energy production, and protein translation, but enhanced stringent response, and harmful substance discharging, and thereby cell death.

#### 2.6.2. The Major Changed Metabolic Pathways in *S. aureus* ATCC25923

**Figure 6:**
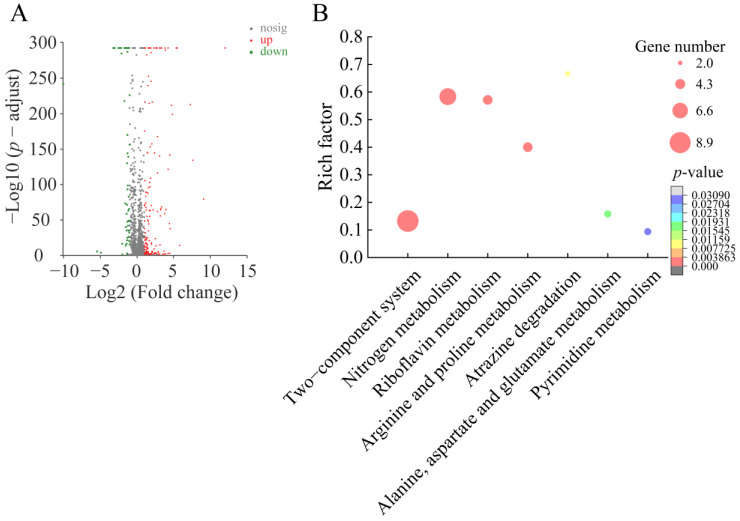
The major changed metabolic pathways in *S. aureus* ATCC25923 triggered by Fragment 1 from *P. kleiniana Wight et Arn*. (**A**) The Volcano plot of the DGEs. (**B**) The significantly altered metabolic pathways in the bacterium.

Approximately 7.3% (196 of 2672 genes) of *S. aureus* ATCC25923 genes were differentially expressed in the treatment group, as compared to the control group. Of these, 156 DEGs showed higher transcriptional levels (fold changes ≥ 2.0), whereas 40 DEGs were significantly down-regulated (fold changes ≤ 0.5) (*p* < 0.05). Based on the comparative transcriptomic analysis, seven significantly altered metabolic pathways were identified in *S. aureus* ATCC25923, including the two-component system; nitrogen metabolism; riboflavin metabolism; arginine and proline metabolism; atrazine degradation; alanine, aspartate and glutamate metabolism; and pyrimidine metabolism (Figure 6, Table 5).

In the arginine and proline metabolism, all the DEGs (*n* = 4) were significantly down-regulated at the transcription levels (0.109-fold to 0.461-fold) in *S. aureus* ATCC25923 (*p* < 0.05). The arginine metabolism converts L-arginine to urea and L-ornithine, which are further metabolized into proline and polyamides that drive collagen synthesis and bioenergetic pathways critical for cell proliferation, respectively [52]. For instance, the DEG (*rocF*, *KQ76_11235*) encoding an arginase was significantly down-regulated (0.461-fold) (*p* < 0.05), and was associated with the ability of *Helicobacter pylori* to establish chronic infections [53].

All the DEGs (*n* = 4) in the riboflavin metabolism were also significantly inhibited (*ribBADEH,* 0.3734-fold to 0.480-fold) (*p* < 0.05). In this pathway, the redox cofactors flavin mononucleotide and flavin adenine dinucleotide and their precursor riboflavin play important roles in many cellular processes, such as respiration, DNA repair, biosyntheses of heme groups, cofactors and nucleotides, fatty acid beta-oxidation, and bioluminescence [54].

Bacteria use two-component signal transduction systems to elicit adaptive responses to environmental changes [55]. In this study, seven DEGs in the two-component system were significantly up-regulated (2.117-fold to 28.924-fold) in *S. aureus* ATCC25923 (*p* < 0.05). For instance, the DEGs (*agrB, KQ76_10520*; and *graS*, *KQ76_03245*) encoding histidine kinases were significantly up-regulated by 2.565-fold and 2.989-fold, respectively (*p* < 0.05). The accessory gene regulator (agr) quorum-sensing system contributes to its pathogenicity of *S. aureus* [56]. GraS, the sensor histidine kinase of the GraXRS system, has been suggested to directly activate the response regulator ArlR [53]. Loss of the ArlR alone impairs the ability of *S. aureus* to respond to host-imposed manganese starvation and glucose limitation [57].

Interestingly, expression of all the DEGs (*n* = 7) in the nitrogen metabolism was significantly increased at the transcription level (3.529-fold to 10.404-fold) in *S. aureus* ATCC25923 (*p* < 0.05). The seven DEGs (*nirBD*, *narHIJZT*) were all involved in nitrate reduction [58,59,60]. Of these, the NirD (*KQ76_12515*) was a small subunit of cytoplasmic NADH-dependent nitrite reductase complex NirBD [61,62]. Over-expression of *nirD* limits RelA-dependent accumulation of guanosine 5′-triphosphate 3′-diphosphate ((p)ppGpp) in vivo and can prevent activation of the stringent response during amino acid starvation in *E. coli* [62].

In the alanine, aspartate, and glutamate metabolism, two DEGs (*carBA*, *KQ76_05770* and *KQ76_05765*) encoding carbamoyl phosphate synthase were significantly up-regulated (2.154-fold and 3.084-fold) in *S. aureus* ATCC25923 (*p* < 0.05). The interface residues located near the CarB region of carboxy phosphate synthetic domain plays a key role in carbamoyl phosphate synthetase, aspartate transcarbamoylase, and dihydroorotase (CAD) complex regulation in the pyrimidine biosynthesis [63]. Correspondingly, in the pyrimidine metabolism, four DEGs (*pyrBCR*, *KQ76_05755*, *KQ76_05760*, and *KQ76_05745*) were also significantly up-regulated (2.968-fold to 3.213-fold) (*p* < 0.05), and encoded an aspartate carbamoyltransferase, a dihydroorotase, and a phosphoribosyl transferase, respectively. The pyrimidines are involved in the synthesis of DNA, RNA, lipids, and carbohydrates. The pyrimidine metabolism is involved in the synthesis, degradation, salvage, interconversion, and transport of these compounds [64].

Taken together, these results indicate that Fragment 1 from *P. kleiniana* Wight et Arn can significantly influence seven metabolic pathways in the Gran-positive *S. aureus* ATCC25923. Of these, the two-component system, alanine, aspartate and glutamate metabolism, and nitrogen metabolism were also changed in the Gram-negative *V. parahaemolyticus* ATCC17802, which led to the enhanced regulation of stringent response in the two pathogens. On the other hand, we also found distinct transcriptomic profiles between the Gram-positive and Gram-negative pathogens triggered by Fragment 1. For example, consistent with the results obtained from the cell structure analysis, *V. parahaemolyticus* ATCC17802 was more sensitive to Fragment 1 treatment, as more metabolic pathways were altered, such as the citrate cycle, glyoxylate and dicarboxylate metabolism, fatty acid degradation, glycine, serine and threonine metabolism, oxidative phosphorylation, pyruvate metabolism, propanoate metabolism, beta-lactam resistance, ABC transporters, PTS, butanoate metabolism, lysine degradation, and QS, which resulted in cell destruction and even death.

In addition, to validate the transcriptome data, we tested 16 representative DEGs (Table S1) via reverse transcription real time-quantitative PCR (RT-qPCR) analysis, and the resulting data were generally correlated with those yielded from the transcriptome analysis (Table S2).

## 3. Materials and Methods 

### 3.1. Bacterial Strains and Culture Conditions

The bacterial strains and culture media used in this study are listed in Table S3. *Vibrio* strains and non-*Vibrio* strains were incubated as described in our recent studies [15,16,65].

### 3.2. Extraction of Bioactive Substances from P. kleiniana Wight et Arn

Fresh *P. kleiniana* Wight et Arn was purchased from the Qian Shan Zhen Pin shop in Guiyang City (26°36′5.01″ N, 106°41′19.90″ E), Guizhou Province, China, in October of 2021. Bioactive substances were extracted from the samples using the methanol and chloroform method described in our recent reports [15,16,66]. Briefly, aliquot of a 500 g of the whole plant sample was lyophilized, pulverised, powded, sonicated, and then filtered and collected for the secondary extraction. The methanol and chloroform phases were separated and then concentrated using the Rotary Evaporator (IKA, Staufen, Germany) [15,16].

### 3.3. Antimicrobial Susceptibility Assay

The susceptibility of the bacterial strains (Table S3) to the extracts from *P. kleiniana* Wight et Arn were determined according to the standard method issued by the Clinical and Laboratory Standards Institute, USA (CLSI, M100-S23, 2018). The antibacterial activity was defined as described previously [15,16]. Broth dilution testing (microdilution) (CLSI, M100-S18, 2018) was used to determine MICs of the extracts. The MIC was defined as described previously [15,16].

### 3.4. Prep-HPLC Analysis

Aliquots of the extracted samples (10 mg/mL) were resolved, centrifuged, filtered, and subjected for the Prep-HPLC Analysis, using Waters 2707 (Waters, Milford, MA, USA) linked with UPLC Sunfire C18 column (5 µm, 10 × 250 mm) (Waters, Milford, MA, USA) with the same parameters and elution conditions described in our recent reports [15,16]. 

### 3.5. UHPLC–MS Analysis

The UHPLC–MS analysis was conducted using the EXIONLC System (Sciex, Framingham, MA, USA) by Shanghai Hoogen Biotech, Shanghai, China [67].

### 3.6. Bacterial Cell Surface Hydrophobicity and Membrane Fluidity Assays

The cell surface hydrophobicity was measured according to the method of Cui et al. [68]. The cell membrane fluidity was measured according to the method of Kuhry et al. [69], using the 1,6-diphenyl-1,3,5-hexatriene (DPH, Sangon, Shanghai, China).

### 3.7. Cell Membrane Permeability Analysis

Cell outer membrane permeability was measured according to the method of Wang et al. [70], with the NPN solution (Sangon, Shanghai, China). The inner membrane permeability was measured according to the method of Huang et al. [71], with the ONPG solution (Sangon, Shanghai, China).

### 3.8. Scanning Electron Microscope (SEM) Assay

The preparation of the samples for the SEM analysis was performed using the method described in our recent reports [15,16,72]. The samples were observed using the Scanning Electron Microscope (Tescan Mira 3 XH, Tescan, Brno, Czech Republic, 5.0 kV, 30,000×).

### 3.9. Illumina RNA Sequencing

The bacterial cell culture at the mid-LGP was treated with Fragment 1 (1× MIC) from *P. kleiniana* Wight et Arn for 6 h, and then collected via centrifugation for the total RNA preparation [15,16,72]. Three independently prepared RNA samples for each strain were subjected for the Illumina RNA sequencing analysis, using Illumina HiSeq 2500 platform (Illumina, Santiago, CA, USA) [72].

### 3.10. RT-qPCR Assay

The RT-qPCR assay was performed according to the method described in our recent reports [15,16,72]. The oligonucleotide primers were designed (Table S1), and synthesized via Sangon (Shanghai, China).

### 3.11. Data Analysis

The DEGs were analyzed as described in our recent reports [15,16,72]. All tests were carried out in triplicate. The data were analyzed using the SPSS statistical analysis software version 17.0 (SPSS Inc., Armonk, NY, USA). One-way analysis of variance (ANOVA) was performed using the least-significant difference (LSD) method and homogeneity of variance test. There was no significant difference between the control and the treatment groups if the generalized *p*-values were more than 0.05; conversely, there was significant difference if *p*-values were less than 0.05.

## 4. Conclusions

In this study, the methanol-phase extract from *P. kleiniana* Wight et Arn showed an inhibition rate of 68.18% against 22 species of common pathogenic bacteria. The methanol-phase extraction inhibited the growth of one species of Gram-positive *S. aureus*, and 14 species of Gram-negative bacteria, including *B. cereus*, *E. cloacae*, *E. coli*, *P. aeruginosa*, *S. typhimurium* 1, *S. dysenteriae*, *S. flexneri*, *S. flexneri*, *S. sonnei*, *V. alginolyticus*, *V. cholerae*, *V. fluvialis*, *V. mimicus*, *V. parahemolyticus*, and *V. vulnificus* strains. This extract was further purified using the Prep-HPLC, and three separated fragments were obtained. Fragment 1 significantly increased bacterial cell surface hydrophobicity and membrane permeability and decreased membrane fluidity, disrupting the cell integrity of the Gram-positive and Gram-negative bacteria such as *S. aureus* ATCC25923, *S. aureus* ATCC8095, *V. parahaemolyticus* ATCC17802, and *V. parahaemolyticus* B5-29. The MIC values of Fragment 1 ranged from 6.25 mg/mL to 50 mg/mL. A total of 66 different compounds in Fragment 1 were identified. The highest relative percentage of the compounds was D-maltose (6.77%), followed by oxymorphone (6.29%), rutin (6.29%), D-proline (5.41%), and L-proline (5.41%). Highly concentrated sugar solutions, such as the D-maltose identified in Fragment 1, are known to be effective antimicrobial agents. The identified oxymorphone and rutin could exert antibacterial activity via damaging the bacterial cell wall and cytoplasmic membrane, respectively. Multiple cellular metabolic pathways altered by Fragment 1 in the representative Gram-negative *V. parahaemolyticus* ATCC17802 and Gram-positive *S. aureus* ATCC25923 pathogens after treatment with Fragment 1 (1× MIC) for 6 h (*p* < 0.05). These results indicated that the energy supply and protein translation of the tested strains was inhibited, the signal transduction was blocked, and the ability to pump foreign harmful substances was reduced, leading to cell death. Overall, the results of this study demonstrate that Fragment 1 from *P. kleiniana* Wight et Arn is a promising candidate for antibacterial medicine and food preservatives.

## Figures and Tables

**Table 1:** Antibacterial activity of crude extracts from *P. kleiniana* Wight et Arn.

Strain	Inhibition Zone (Diameter, mm)		MIC (mg/mL)	
	CPE	MPE	CPE	MPE
*Aeromonas hydrophila* ATCC35654	-	-	-	-
*Bacillus cereus* A1-1	7.03 ± 0.01	10.54 ± 0.48	50	6.25
*Bacillus cereus* A2-2	7.11 ± 0.02	10.54 ± 0.75	50	1.56
*Enterobacter cloacae* ATCC13047	7.00 ± 0.11	7.11 ± 0.26	50	50
*Enterobacter cloacae* C1-1	-	-	-	-
*Escherichia coli* ATCC8739	-	7.62 ± 0.37	-	25
*Escherichia coli* ATCC25922	-	-	-	-
*Escherichia coli* K12	-	7.51 ± 0.29	-	25
*Enterobacter sakazakii* CMCC45401	-	-	-	-
*Klebsiella pneumoniae* 8-2-10-8	-	-	-	-
*Klebsiella pneumoniae* 8-2-1-14	-	-	-	-
*Pseudomonas aeruginosa* ATCC9027	-	10.51 ± 0.41	-	6.25
*Pseudomonas aeruginosa* ATCC27853	-	8.14 ± 0.32	-	25
*Salmonella enterica* subsp. *enterica* (*ex* Kauffmann and Edwards) Le Minor and Popoff serovar Choleraesuis ATCC13312	-	-	-	-
*Salmonella paratyphi-A* CMCC50093	-	-	-	-
*Salmonellaenterica* subsp. *enterica* (*ex* Kauffmann and Edwards) Le Minor and Popoff serovar Vellore ATCC15611	7.09 ± 0.09	10.11 ± 0.61	50	6.25
*Salmonella* E1-1	-	-	-	-
*Shigella dysenteriae* CMCC51252	7.02 ± 0.11	9.29 ± 0.51	50	12.5
*Shigella flexneri* CMCC51572	7.82 ± 0.20	10.17 ± 0.20	25	6.25
*Shigella flexneri* ATCC12022	-	-	-	-
*Shigella flexneri* CMCC51574	-	9.17 ± 0.21	-	12.5
*Shigella sonnei* ATCC25931	7.00 ± 0.11	8.19 ± 0.51	50	25
*Shigella sonnet* CMCC51592	-	-	-	-
*Staphylococcus aureus* ATCC25923	7.03 ± 0.14	9.41 ± 0.27	50	12.5
*Staphylococcus aureus* ATCC8095	7.07 ± 0.15	10.15 ± 0.24	50	6.25
*Staphylococcus aureus* ATCC29213	7.78 ± 0.10	9.21 ± 0.01	25	12.5
*Staphylococcus aureus* ATCC6538	7.62 ± 0.61	9.55 ± 0.37	25	12.5
*Staphylococcus aureus* D1-1	7.11 ± 0.25	7.00 ± 0.51	50	50
*Vibrio alginolyticus* ATCC17749	-	10.11 ± 0.24	-	3.13
*Vibrio alginolyticus* ATCC33787	-	-	-	-
*Vibrio cholerae* GIM1.449	-	7.00 ± 0.14	-	50
*Vibrio cholerae* Q10-54	7.22 ± 0.10	7.02 ± 0.21	50	50
*Vibrio fluvialis* ATCC33809	-	7.12 ± 0.03	-	50
*Vibrio harvey* ATCC BAA-1117	-	-	-	-
*Vibrio harveyi* ATCC33842	-	-	-	-
*Vibrio mimicus* bio-56759	7.21 ± 0.41	11.00 ± 0.32	25	3.13
*Vibrio parahemolyticus* ATCC17802	-	10.67 ± 1.21	-	1.56
*Vibrio parahemolyticus* ATCC33847	8.63 ± 0.24	7.14 ± 0.12	12.5	50
*Vibrio parahemolyticus* B3-13	7.17 ± 0.29	12.33 ± 0.65	50	3.13
*Vibrio parahemolyticus* B4-10	-	11.26 ± 0.34	-	6.25
*Vibrio parahemolyticus* B5-29	7.17 ± 0.04	13.77 ± 0.85	50	3.13
*Vibrio parahemolyticus* B9-35	7.20 ± 0.09	13.15 ± 0.44	25	3.13
*Vibrio parahemolyticus* A1-1	7.13 ± 0.15	10.35 ± 0.58	50	3.13
*Vibrio vulnificus* ATCC27562	7.65 ± 0.44	7.01 ± 0.23	25	50

Note: CPE: chloroform-phase extract. MPE: methanol-phase extract. -: no bacteriostasis activity. Inhibition zone: diameter includes the disk diameter (6 mm). MIC: minimum inhibitory concentration. Values were means ± standard deviation (S.D.) of three parallel measurements.

**Table 2:** Antibacterial activity of Fragment 1 of the methanol-phase extract from *P. kleiniana* Wight et Arn.

Strain	Inhibition Zone (Diameter, mm)	MIC (mg/mL)
*B. cereus* A2-2	8.03 ± 0.45	6.25
*S. flexneri* CMCC51572	7.50 ± 0.50	6.25
*S. aureus* ATCC25923	8.03 ± 0.40	12.5
*S. aureus* ATCC8095	9.53 ± 0.35	6.25
*S. aureus* ATCC6538	7.10 ± 0.36	50.0
*V. parahemolyticus* ATCC17802	10.31 ± 0.62	6.25
*V. parahemolyticus* A1-1	8.57 ± 0.60	25.0
*V. parahemolyticus* B3-13	10.37 ± 0.32	6.25
*V. parahemolyticus* B4-10	10.30 ± 0.50	12.5
*V. parahemolyticus* B5-29	11.30 ± 0.26	6.25
*V. parahemolyticus* B9-35	11.27 ± 0.40	12.5

**Table 3:** Compounds identified in Fragment 1 from *P. kleiniana* Wight et Arn via UHPLC–MS analysis.

Peak No.	Identified Compound	Compound Nature	Rt (min)	Formula	Exact Mass	Peak Area (%)
1	D-Maltose	Carbohydrates	0.76	C_12_H_22_O_11_	342.1162	6.77%
2	Oxymorphone	Phenanthrenes and derivatives	11.18	C_17_H_19_NO_4_	301.1314	6.29%
3	Rutin	Flavonoids	12.99	C_27_H_30_O_16_	281.0899	6.29%
4	D-Proline	Amino acid and derivatives	0.76	C_5_H_9_NO_2_	115.0633	5.41%
5	L-Proline	Amino acid and derivatives	0.73	C_5_H_9_NO_2_	115.0633	5.41%
6	L-Glutamic acid	Amino acid and derivatives	0.66	C_5_H_9_NO_4_	147.0532	5.20%
7	Sucrose	Carbohydrates	0.89	C_12_H_22_O_11_	342.1162	3.62%
8	Cynaroside	Flavonoids	12.98	C_21_H_20_O_11_	282.162	3.37%
9	Piperlonguminine	Alkaloids	10.57	C_16_H_19_NO_3_	273.1365	3.21%
10	5-Aminovaleric acid	Amino acid and derivatives	1.11	C_5_H_11_NO_2_	117.079	3.12%
11	D-Glutamine	Carboxylic acids and derivatives	0.66	C_5_H_10_N_2_O_3_	146.0691	2.99%
12	L-Lysine	Amino acid and derivatives	0.64	C_6_H_14_N_2_O_2_	146.1055	2.99%
13	p-Octopamine	Phenols	3.84	C_8_H_11_NO_2_	153.079	2.96%
14	Oleic acid	Fatty acyls	13.03	C_18_H_34_O_2_	282.2559	2.91%
15	Isoquercitrin	Flavonoids	10.58	C_21_H_20_O_12_	274.1933	2.44%
16	L-Pipecolic acid	Amino acid and derivatives	0.69	C_6_H_11_NO_2_	129.079	2.31%
17	Moracin C	Phenols	0.67	C_19_H_18_O_4_	129.0426	2.31%
18	Kojibiose	Fatty acyls	0.72	C_12_H_22_O_11_	342.1162	2.22%
19	Gluconic acid	Carbohydrates	0.69	C_6_H_12_O_7_	196.0583	1.97%
20	Betaine	Alkaloids	1.06	C_5_H_11_NO_2_	117.079	1.51%
21	L-Valine	Amino acid and derivatives	0.93	C_5_H_11_NO_2_	117.079	1.49%
22	D-alpha-Aminobutyric acid	Carboxylic acids and derivatives	0.65	C_4_H_9_NO_2_	103.0633	1.46%
23	cis-Aconitic acid	Organic acids and derivatives	1.46	C_6_H_6_O_6_	174.0164	1.34%
24	Lactulose	Organooxygen compounds	0.77	C_12_H_22_O_11_	342.1162	1.33%
25	Turanose	Fatty acyls	0.79	C_12_H_22_O_11_	342.1162	1.33%
26	L-Pipecolic acid	Amino acid and derivatives	1.47	C_6_H_11_NO_2_	129.079	1.15%
27	DL-Norvaline	Amino acid and derivatives	1.05	C_5_H_11_NO_2_	117.079	1.11%
28	L-Asparagine	Amino acid and derivatives	0.64	C_4_H_8_N_2_O_3_	132.0535	1.11%
29	Malic acid	Hydroxy acids and derivatives	0.8	C_4_H_6_O_5_	134.0215	0.90%
30	Trigonelline	Alkaloids	0.82	C_7_H_7_NO_2_	137.0477	0.90%
31	Acetamide	Alkaloids	13.95	C_2_H_5_NO	59.03711	0.88%
32	Beta-D-fructose 2-phosphate	Organooxygen compounds	0.75	C_6_H_13_O_9_P	260.0297	0.77%
33	22-Dehydroclerosterol	Steroids	12.59	C_29_H_46_O	410.3549	0.76%
34	Artemisinin	Sesquiterpenoids	13.02	C_15_H_22_O_5_	282.1467	0.72%
35	Kaempferol-3-O-rutinoside	flavonoids	6.29	C_27_H_30_O_15_	594.1585	0.54%
36	L-Homoserine	Amino acid and derivatives	0.67	C_4_H_9_NO_3_	119.0582	0.52%
37	L-Threonine	Amino acid and derivatives	0.64	C_4_H_9_NO_3_	119.0582	0.50%
38	Palmitic acid	Lipids	12.92	C_16_H_32_O_2_	256.2402	0.49%
39	O-Acetylethanolamine	Alkaloids	0.67	C_4_H_9_NO_2_	103.0633	0.46%
40	Galactose 1-phosphate	Organooxygen compounds	0.65	C_6_H_13_O_9_P	260.0297	0.46%
41	Glucose 1-phosphate	Organooxygen compounds	13	C_6_H_13_O_9_P	260.0297	0.45%
42	Adenosine 5′-monophosphate	Nucleotide and its derivates	1.38	C_10_H_14_N_5_O_7_P	347.0631	0.43%
43	L-Arginine	Amino acid and derivatives	0.6	C_6_H_14_N_4_O_2_	174.1117	0.43%
44	Maltotriose	Organooxygen compounds	1.23	C_18_H_32_O_16_	504.169	0.40%
45	Indole	Alkaloids	3.82	C_8_H_7_N	117.0578	0.38%
46	D-Glucose 6-phosphate	Carbohydrates	0.65	C_6_H_13_O_9_P	260.0297	0.37%
47	D-Aspartic acid	Alkaloids	0.76	C_4_H_7_NO_4_	133.0375	0.36%
48	Vitexin rhamnoside	Flavonoids	6.78	C_27_H_30_O_14_	578.1636	0.35%
49	L-Aspartic acid	Amino acid and derivatives	0.63	C_4_H_7_NO_4_	133.0375	0.33%
50	Maltol	Flavonoids	0.9	C_6_H_6_O_3_	126.0317	0.33%
51	Astragalin	Flavonoids	6.52	C_21_H_20_O_11_	448.1006	0.32%
52	3-Hydroxy-3-methylpentane-1,5-dioic acid	Amino acid and derivatives	2.32	C_6_H_10_O_5_	162.0528	0.31%
53	Campesterol	Steroids and steroid derivatives	12.18	C_28_H_48_O	400.3705	0.30%
54	L-Ornithine	Amino acid and derivatives	0.55	C_5_H_12_N_2_O_2_	132.0899	0.30%
55	Adenosine	Nucleotide and its derivates	2.58	C_10_H_13_N_5_O_4_	267.0968	0.29%
56	Vidarabine	Purine nucleosides	2.28	C_10_H_13_N_5_O_4_	267.0968	0.27%
57	Nicotinic acid	Nicotinic acid derivatives	0.73	C_6_H_5_NO_2_	123.032	0.27%
58	Pelargonidin-3-O-glucoside	Flavonoids	1.11	C_21_H_20_O_1_0	100.0524	0.26%
59	L-Citruline	Amino acid and derivatives	0.66	C_6_H_13_N_3_O_3_	175.0957	0.26%
60	Diallyl disulfide	Miscellaneous	0.68	C_6_H_10_S_2_	146.0224	0.26%
61	Sarracine	Alkaloids	13.14	C_18_H_27_NO_5_	337.1889	0.22%
62	N-Acetylputrescine	Phenolamides	1.79	C_6_H_14_N_2_O	130.1106	0.22%
63	Salicylic acid	Organic acid	7.06	C_7_H_6_O_3_	138.0317	0.22%
64	5-Methylcytosine	Nucleotide and its derivates	2.26	C_5_H_7_N_3_O	125.0589	0.21%
65	Ellagic acid	Phenols	6.12	C_14_H_6_O_8_	302.0063	0.21%
66	Isodiospyrin	Quinones	11.28	C_22_H_14_O_6_	374.079	0.21%

**Table 4:** The major altered metabolic pathways in *V. parahaemolyticus* ATCC17802.

Metabolic Pathway	Gene ID	Gene Name	Fold Change	Gene Description
Citrate cycle	*WU75_19785*	*sucA*	0.146	2-oxoglutarate dehydrogenase
	*WU75_07425*	*pckA*	0.465	Phosphoenolpyruvate carboxykinase
	*WU75_19790*	*sucB*	0.133	Dihydrolipoamide succinyltransferase
	*WU75_11550*	*acnB*	0.143	Bifunctional aconitate hydratase 2/2-methylisocitrate dehydratase
	*WU75_19795*	*sucC*	0.134	Succinyl-CoA synthetase subunit beta
	*WU75_19800*	*sucD*	0.16	Succinyl-CoA synthetase subunit alpha
	*WU75_19770*	*sdhD*	0.199	Succinate dehydrogenase
	*WU75_19780*	*sdhB*	0.157	Succinate dehydrogenase
	*WU75_19765*	*sdhC*	0.182	Succinate dehydrogenase
	*WU75_13785*	*fumA*	0.497	Fumarate hydratase
	*WU75_09605*	*icd*	0.179	Isocitrate dehydrogenase
	*WU75_19775*	*sdhA*	0.144	Succinate dehydrogenase
	*WU75_06430*	*mdh*	0.177	Malate dehydrogenase
	*WU75_16530*	*lpd*	0.35	Dihydrolipoamide dehydrogenase
Glyoxylate and dicarboxylate metabolism	*WU75_19760*	*gltA*	0.129	Type II citrate synthase
	*WU75_19150*	*aceA*	0.37	Isocitrate lyase
	*WU75_19145*	*aceB*	0.352	Malate synthase
	*WU75_00290*	*aceB*	0.315	Malate synthase
	*WU75_10840*	*phbB*	0.277	3-ketoacyl-ACP reductase
	*WU75_03265*	*katE*	2.389	Catalase
Fatty acid degradation	*WU75_22235*	*fadB*	0.151	Multifunctional fatty acid oxidation complex subunit alpha
	*WU75_08655*	*fadE*	0.184	Acyl-CoA dehydrogenase
	*WU75_20175*	*fadJ*	0.204	Multifunctional fatty acid oxidation complex subunit alpha
	*WU75_22230*	*fadA*	0.208	3-ketoacyl-CoA thiolase
	*WU75_20180*	*fadA*	0.305	3-ketoacyl-CoA thiolase
	*WU75_10835*	*atoB*	0.433	Acetyl-CoA acetyltransferase
	*WU75_10445*	*atoB*	0.445	Acetyl-CoA acetyltransferase
	*WU75_12560*	*fadE*	0.452	Acyl-CoA dehydrogenase
	*WU75_19885*	*fadD*	0.493	Long-chain fatty acid—CoA ligase
Glycine, serine and threonine metabolism	*WU75_14910*	*gcvP*	0.113	Glycine dehydrogenase
	*WU75_14915*	*gcvH*	0.127	Glycine cleavage system protein H
	*WU75_10395*	*betA*	0.162	Choline dehydrogenase
	*WU75_14930*	*gcvT*	0.184	Glycine cleavage system protein T
	*WU75_16130*	*lysC*	0.187	Aspartate kinase
	*WU75_14920*	*glyA*	0.203	Serine hydroxymethyltransferase
	*WU75_16140*	*ectB*	0.222	Diaminobutyrate-2-oxoglutarate aminotransferase
	*WU75_16145*	*ectA*	0.246	L-2,4-diaminobutyric acid acetyltransferase
	*WU75_10400*	*betB*	0.259	Betaine-aldehyde dehydrogenase
	*WU75_00565*	*sdaA*	0.264	Serine dehydratase
	*WU75_16135*	*ectC*	0.27	Ectoine synthase
	*WU75_02030*	*trpB*	0.397	Tryptophan synthase subunit beta
	*WU75_05755*	*thrC*	0.429	Threonine synthase
	*WU75_05760*	*thrB*	0.47	Serine kinase
	*WU75_05330*	*glxK*	0.495	Glycerate kinase
Oxidative phosphorylation	*WU75_06010*	*petC*	0.195	Cytochrome C
	*WU75_06015*	*petB*	0.209	Cytochrome B
	*WU75_14570*	*ccoO*	0.228	Peptidase S41
	*WU75_14575*	*ccoN*	0.272	Cbb3-type cytochrome c oxidase subunit I
	*WU75_14560*	*ccoP*	0.301	Cytochrome Cbb3
	*WU75_06485*	*ppa*	0.339	Inorganic pyrophosphatase
	*WU75_06020*	*petA*	0.442	Ubiquinol-cytochrome C reductase
	*WU75_14565*	*ccoQ*	0.475	Cytochrome C oxidase
	*WU75_02240*	*cyoC*	0.478	Cytochrome o ubiquinol oxidase subunit III
	*WU75_19125*	*ppk2*	2.159	Polyphosphate kinase
	*WU75_09420*	*cydA*	3.637	Cytochrome d terminal oxidase subunit 1
	*WU75_09415*	*cydB*	4.11	Cytochrome d ubiquinol oxidase subunit 2
	*WU75_09410*	*cydX*	5.362	Membrane protein
Pyruvate metabolism	*WU75_01940*	*yiaY*	0.171	Alcohol dehydrogenase
	*WU75_03655*	*lldD*	0.276	Lactate dehydrogenase
	*WU75_22155*	*dld*	0.322	Lactate dehydrogenase
	*WU75_16665*	*oadA*	0.324	Oxaloacetate decarboxylase
	*WU75_16060*	*aldB*	0.397	Aldehyde dehydrogenase
	*WU75_20855*	*gloA*	2.451	Lactoylglutathione lyase
	*WU75_12805*	*pta*	8.464	Phosphate acetyltransferase
	*WU75_02150*	*ackA*	8.851	Acetate kinase
	*WU75_12810*	*ackA*	10.365	Acetate kinase
	*WU75_09685*	*pflD*	12.853	Pyruvate formate-lyase
	*WU75_00810*	*gloA*	13.536	Glyoxalase
Propanoate metabolism	*WU75_15760*	*prpF*	0.402	3-methylitaconate isomerase
	*WU75_15770*	*prpC*	0.435	Methylcitrate synthase
beta-Lactam resistance	*WU75_09315*	*acrA*	6.699	Hemolysin D
	*WU75_09310*	*acrB*	8.911	Multidrug transporter
	*WU75_09925*	*acrA*	40.366	Hemolysin D
ABC transporters	*WU75_10385*	*proW*	0.106	ABC transporter permease
	*WU75_16175*	*proX*	0.116	Glycine/betaine ABC transporter substrate-binding protein
	*WU75_10390*	*proX*	0.122	Glycine/betaine ABC transporter substrate-binding protein
	*WU75_12775*	*oppC*	0.133	Peptide ABC transporter permease
	*WU75_10380*	*proV*	0.138	ABC transporter ATP-binding protein
	*WU75_09655*	*aotM*	0.143	Amino acid ABC transporter permease
	*WU75_09665*	*aotJ*	0.144	Nickel transporter
	*WU75_13090*	*yejA*	0.151	Diguanylate cyclase
	*WU75_12770*	*oppB*	0.164	Oligopeptide transporter permease
	*WU75_12780*	*oppD*	0.172	Oligopeptide transporter ATP-binding component
	*WU75_09660*	*aotQ*	0.176	ABC transporter
	*WU75_16170*	*proW*	0.199	Glycine/betaine ABC transporter permease
	*WU75_08085*	*oppA*	0.201	Peptide ABC transporter substrate-binding protein
	*WU75_07210*	*yejA*	0.204	Diguanylate cyclase
	*WU75_12765*	*oppA*	0.214	Peptide ABC transporter substrate-binding protein
	*WU75_07220*	*yejB*	0.22	Hypothetical protein
	*WU75_07215*	*yejE*	0.221	Peptide ABC transporter permease
	*WU75_09670*	*aotP*	0.228	Amino acid transporter
	*WU75_12785*	*oppF*	0.228	Peptide ABC transporter ATP-binding protein
	*WU75_04720*	*oppA*	0.341	Peptide ABC transporter substrate-binding protein
	*WU75_16165*	*proV*	0.343	Glycine/betaine ABC transporter ATP-binding protein
	*WU75_14765*	*aapQ*	0.377	Amino acid ABC transporter permease
	*WU75_03180*	*malE*	0.4	Sugar ABC transporter substrate-binding protein
	*WU75_14775*	*aapP*	0.405	ABC transporter ATP-binding protein
	*WU75_04605*	*vcaM*	0.406	Multidrug ABC transporter ATP-binding protein
	*WU75_14055*	*mdlB*	0.411	Multidrug ABC transporter ATP-binding protein
	*WU75_10275*	*rbsD*	0.438	D-ribose pyranase
	*WU75_05845*	*btuF*	0.487	Vitamin B12-binding protein
	*WU75_14760*	*aapJ*	0.491	Amino acid ABC transporter substrate-binding protein
	*WU75_03185*	*malK*	2.175	Maltose/maltodextrin transporter ATP-binding protein
	*WU75_19815*	*znuA*	2.204	Zinc ABC transporter substrate-binding protein
	*WU75_19810*	*znuC*	2.491	Zinc ABC transporter ATPase
	*WU75_02265*	*artP*	2.617	Arginine ABC transporter ATP-binding protein
	*WU75_19805*	*znuB*	2.666	Membrane protein
	*WU75_00425*	*macB*	14.353	Macrolide transporter
Two-component system	*WU75_07480*	*glnG*	0.186	Nitrogen regulation protein NR(I)
	*WU75_13735*	*mcp*	0.218	Chemotaxis protein
	*WU75_15795*	*tctB*	0.237	TctB
	*WU75_21750*	*dctD*	0.288	C4-dicarboxylate ABC transporter
	*WU75_13155*	*ttrB*	0.31	4Fe-4S ferredoxin
	*WU75_21770*	*dctP*	0.31	C4-dicarboxylate ABC transporter
	*WU75_01920*	*mcp*	0.32	Chemotaxis protein
	*WU75_21745*	*dctB*	0.352	ATPase
	*WU75_10200*	*phoA*	0.353	Alkaline phosphatase
	*WU75_21765*	*dctQ*	0.368	C4-dicarboxylate ABC transporter permease
	*WU75_00210*	*dctD*	0.406	C4-dicarboxylate ABC transporter
	*WU75_16210*	*qseC*	0.423	Histidine kinase
	*WU75_23015*	*fliC*	0.435	Flagellin
	*WU75_07100*	*mcp*	0.453	Chemotaxis protein
	*WU75_13380*	*crp*	0.457	Transcriptional regulator
	*WU75_09825*	*mcp*	0.471	Chemotaxis protein
	*WU75_16525*	*hapR*	0.477	LuxR family transcriptional regulator
	*WU75_15800*	*tctA*	0.485	Tripartite tricarboxylate transporter TctA
	*WU75_14800*	*mcp*	0.491	Chemotaxis protein
	*WU75_06085*	*tolC*	2.068	Outer membrane channel protein
	*WU75_15630*	*dcuB*	2.125	C4-dicarboxylate transporter
	*WU75_06045*	*degP*	2.148	Serine endoprotease DegQ
	*WU75_04355*	*mcp*	2.163	Chemotaxis protein
	*WU75_10915*	*luxQ*	3.377	ATPase
	*WU75_22175*	*mcp*	4.001	Chemotaxis protein
	*WU75_02450*	*pfeR*	4.828	Transcriptional regulator
	*WU75_18570*	*cpxA*	10.981	Two-component sensor protein
	*WU75_18575*	*cpxR*	26.5	Transcriptional regulator
Alanine, aspartate and glutamate metabolism	*WU75_06265*	*glmS*	0.037	Glucosamine-fructose-6-phosphate Aminotransferase
	*WU75_07465*	*glnA*	0.123	Glutamine synthetase
	*WU75_04655*	*putA*	0.145	Pyrroline-5-carboxylate dehydrogenase
	*WU75_14680*	*-*	0.286	NAD-glutamate dehydrogenase
	*WU75_05875*	*carB*	0.343	Carbamoyl phosphate synthase large subunit
	*WU75_05820*	*gltB*	0.414	Glutamate synthase
	*WU75_05825*	*gltD*	0.44	Glutamate synthase
	*WU75_05880*	*carA*	0.46	Carbamoyl phosphate synthase small subunit
	*WU75_18095*	*pyrI*	0.462	Aspartate carbamoyltransferase regulatory subunit
	*WU75_18090*	*pyrB*	0.466	Aspartate carbamoyltransferase catalytic subunit
	*WU75_20915*	*ansA*	2.141	Cytoplasmic asparaginase I
	*WU75_01110*	*ansB*	2.718	L-asparaginase II
	*WU75_18550*	*aspA*	7.015	Aspartate ammonia-lyase
PTS	*WU75_03285*	*ptsN*	0.462	PTS fructose transporter subunit IIA
	*WU75_12990*	*ptsG*	0.5	PTS glucose transporter subunit IIBC
	*WU75_17910*	*celC*	2.36	Molecular chaperone TorD
	*WU75_14970*	*fruB*	2.451	Bifunctional PTS system fructose-Specific transporter subunit IIA/HPr protein
	*WU75_19555*	*ptsH*	3.973	PTS sugar transporter
	*WU75_00455*	*ulaB*	3.977	PTS ascorbate transporter subunit IIB
	*WU75_19550*	*ptsI*	4.075	Phosphoenolpyruvate-protein Phosphotransferase
	*WU75_00460*	*cmtB*	4.118	PTS system mannitol-specific Transporter subunit IIA
	*WU75_01640*	*cmtB*	4.539	PTS mannitol transporter subunit IIA
	*WU75_14960*	*fruA*	5.096	PTS fructose transporter subunit IIBC
	*WU75_00450*	*ulaA*	6.946	PTS beta-glucoside transporter subunit IIBC
Butanoate metabolism	*WU75_01985*	*acsA*	0.334	Acetoacetyl-CoA synthetase
	*WU75_10825*	*phaC*	0.336	Poly(3-hydroxyalkanoate) synthetase
Lysine degradation	*WU75_21960*	*ldcC*	7.207	Lysine decarboxylase LdcC
QS	*WU75_07805*	*-*	0.109	Cytochrome C
	*WU75_07800*	*-*	0.181	ABC transporter permease
	*WU75_07795*	*-*	0.202	ABC transporter permease
	*WU75_07810*	*ddpD*	0.216	ABC transporter ATP-binding protein
	*WU75_11620*	*-*	0.218	Peptide ABC transporter permease
	*WU75_11630*	*-*	0.233	Peptide ABC transporter substrate-binding protein
	*WU75_11625*	*-*	0.261	Peptide ABC transporter permease
	*WU75_11610*	*ddpF*	0.358	Chemotaxis protein
	*WU75_11615*	*ddpD*	0.484	Sugar ABC transporter ATP-binding protein
	*WU75_21410*	*aphA*	2.288	Transcriptional regulator
Nitrogen metabolism	*WU75_00760*	*ncd2*	0.276	2-nitropropane dioxygenase
	*WU75_10810*	*napA*	2.286	Nitrate reductase
	*WU75_15655*	*nirD*	3.934	Nitrite reductase
	*WU75_10815*	*napB*	6.27	Nitrate reductase
	*WU75_08850*	*hcp*	63.107	Hydroxylamine reductase

**Table 5:** The major altered metabolic pathways in *S. aureus* ATCC25923.

Metabolic Pathway	Gene ID	Gene Name	Fold Change	Gene Description
Two-component system	*KQ76_00500*	-	0.373	Capsular biosynthesis protein
	*KQ76_00560*	*wecC*	0.490	UDP-N-acetyl-D-mannosamine dehydrogenase
	*KQ76_12475*	*nreC*	2.117	Nitrate respiration regulation response regulator NreC
	*KQ76_12480*	*nreB*	2.276	Nitrate respiration regulation sensor histidine kinase NreB
	*KQ76_12485*	*nreA*	2.433	Nitrate respiration regulation accessory nitrate sensor NreA
	*KQ76_10520*	*agrB*	2.565	Histidine kinase
	*KQ76_03245*	*graS*	2.989	Histidine kinase
	*KQ76_10785*	*kdpF*	5.371	ATPase
	*KQ76_04230*	*dltC*	28.924	Alanine-phosphoribitol ligase
Nitrogen metabolism	*KQ76_12490*	*narI*	3.529	Nitrate reductase
	*KQ76_12515*	*nirD*	4.199	Nitrite reductase
	*KQ76_12520*	*nirB*	5.060	Nitrite reductase
	*KQ76_12460*	*narT*	6.376	Nitrate transporter NarT
	*KQ76_12500*	*narH*	5.799	Nitrate reductase
	*KQ76_12505*	*narZ*	8.442	Nitrate reductase
	*KQ76_12495*	*narJ*	10.404	Nitrate reductase
Riboflavin metabolism	*KQ76_09200*	*ribE*	0.373	Riboflavin synthase subunit alpha
	*KQ76_09195*	*ribBA*	0.413	GTP cyclohydrolase
	*KQ76_09205*	*ribD*	0.430	Diaminohydroxyphosphoribosylaminopyrimidine deaminase
	*KQ76_09190*	*ribH*	0.480	6,7-dimethyl-8-ribityllumazine synthase
Arginine and proline metabolism	*KQ76_09185*	*fadM*	0.109	Proline dehydrogenase
	*KQ76_00580*	*-*	0.218	Aldehyde dehydrogenase
	*KQ76_13360*	*-*	0.303	1-pyrroline-5-carboxylate dehydrogenase
	*KQ76_11235*	*rocF*	0.461	Arginase
Atrazine degradation	*KQ76_11915*	*ureC*	0.406	Urease subunit alpha
	*KQ76_11910*	*ureB*	0.412	Urease subunit beta
Alanine, aspartate and glutamate metabolism	*KQ76_13360*	-	0.303	1-pyrroline-5-carboxylate dehydrogenase
	*KQ76_05770*	*carB*	2.158	Carbamoyl phosphate synthase large subunit
	*KQ76_05765*	*carA*	3.084	Carbamoyl phosphate synthase small subunit
Pyrimidine metabolism	*KQ76_05745*	*pyrR*	2.968	Phosphoribosyl transferase
	*KQ76_05760*	*pyrC*	3.115	Dihydroorotase
	*KQ76_05755*	*pyrB*	3.213	Aspartate carbamoyltransferase

## Data Availability

Data is contained within the article or Supplementary Materials. The complete lists of DEGs in the two strains are available in the NCBI SRA database (https://submit.ncbi.nlm.nih.gov/subs/bioproject/, accessed on 29 November 2022) under the accession number PRJNA906658.

## Supplementary Materials

The following supporting information can be downloaded at: https://www.mdpi.com/article/10.3390/foods12081640/s1, Table S1: The oligonucleotide primers designed and used in the RT-qPCR assay; Table S2: The relative expression of representative DEGs by the RT-qPCR assay; Table S3: The bacterial strains and media used in this study; Figure S1: The Prep−HPLC diagram of purifying the methanol-phase crude extract from *P. kleiniana* Wight et Arn.
