# Supplementary material for: Identification of Antibacterial Components and Modes in the Methanol-Phase Extract from a Herbal Plant Potentilla kleiniana Wight et Arn
Source: Foods. 2023 Apr 13;12(8):1640. doi: 10.3390/foods12081640 (PMC10137656; doi:10.3390/foods12081640)
Supplement: Supplementary file 1 [file foods-12-01640-s001.zip › foods-2291881-supplementary.pdf]

plants

MDPI

Supplementary Materials

Table S1. The oligonucleotide primers designed and used in the RT-qPCR assay.

Strain	Primer	Sequence (5′->3′)
S. aureus ATCC25923	*KQ76_04230*-F	GGTATTATTGATTCTTTCCAAAC
	KQ76_04230-R	TCGTAACTCTTCTAATGCTTC
	KQ76_12495-F	GTACGAGATGTTTGGACTAGA
	KQ76_12495-R	GCATGATGTTTCACTCCTTTT
	KQ76_05765-F	GAAGTTTGGTCATCGTGGTG
	KQ76_05765-R	ATCTGACGGTCCTGGATTTG
	KQ76_05755-F	ATTGATTTGTGGCGACATT
	KQ76_05755-R	AATCCAAGCATTTGGGCTA
	KQ76_09185-F	CCTTATGGCGATGATTGGT
	KQ76_09185-R	TAAGCCAGCAGGTTTCACA
	KQ76_11910-F	AAAATACAGGAGATCGACCAA
	KQ76_11910-R	CCATACCACGAAAACCAAA
	KQ76_09200-F	CGGTAGGTTTGGTGGACAT
	KQ76_09200-R	ACGCCTCGTTTCTGGAATA
	KQ76_00500-F	CACATTTCATCGGTTCAGA
	KQ76_00500-R	CAACAACTAACTTCGCATTA
V. parahaemolyticus ATCC17802	*WU75_19785*-F	GATGACAGGTGAGAAGGCTAT
	WU75_19785-R	GTCCTGACCAGAAATACGG
	WU75_00290-F	TATCGCTGAAGCCGTATTT
	WU75_00290-R	ACATGGCGGTAATCAATCA
	WU75_20180-F	GTGAGCGTGTTGCTATTGTC
	WU75_20180-R	GTAAGCGTCGGTATGGATG
	WU75_22235-F	CCAGGCTTTGGTGGTTGTG
	WU75_22235-R	TGAGTGTCAGTGGCGAGGT
	WU75_16140-F	CTCGCCGCACTGGATAACT
	WU75_16140-R	CGCTGCTTCTGCCTTTCAC
	WU75_08850-F	GGCGTGGTTAGGCACAGAT
	WU75_08850-R	GCCGAAGGTCGTAGTTTCA
	WU75_10815-F	AGTGTCACTGAACGCAAAC
	WU75_10815-R	CAGAAGTAACGACGAGGAG
	WU75_14960-F	TTGTTGCGACGCTATTTACG
	WU75_14960-R	GCTGTCTGAACCCATGTTGT

Table S2. The relative expression of the representative DEGs by the RT-qPCR assay.

**Sample**	**Gene**	**Predicted protein**	**Fold change**	
			**RNA-Seq.**	**RT-PCR**
S. aureus ATCC25923	*KQ76_04230*	Alanine-phosphoribitol ligase	28.924	29.731
	KQ76_12495	Nitrate reductase	10.404	8.465
	KQ76_05765	Carbamoyl phosphate synthase small subunit	3.084	6.324
	KQ76_05755	Aspartate carbamoyltransferase	3.213	12.109
	KQ76_09185	Proline dehydrogenase	0.109	0.101
	KQ76_11910	Urease subunit beta	0.412	0.472
	KQ76_09200	Riboflavin synthase subunit alpha	0.373	1.237
	KQ76_00500	Capsular biosynthesis protein	0.373	0.369
V. parahaemolyticus ATCC17802	*WU75_19785*	2-oxoglutarate dehydrogenase	0.146	0.475
	WU75_00290	Malate synthase	0.315	0.331
	WU75_20180	3-ketoacyl-CoA thiolase	0.305	0.643
	WU75_22235	Multifunctional fatty acid oxidation complex subunit alpha	0.151	0.149
	WU75_16140	Diaminobutyrate--2-oxoglutarate aminotransferase	0.222	25.317
	WU75_08850	Hydroxylamine reductase	63.107	59.973
	WU75_10815	Nitrate reductase	6.27	6.86
	WU75_14960	PTS fructose transporter subunit IIBC	5.096	5.755

Table S3. Bacterial strains and media used in this study

Bacterial Strain	Culture Medium	Source
Aeromonas hydrophila ATCC35654	TSB	ATCC, United States
Bacillus cereus A1-1	TSB	LS-SHOU, China
Bacillus cereus A2-	TSB	LS-SHOU, China
Enterobacter cloacae ATCC13047	TSB	Biobw, China
Enterobacter cloacae C1-1	LB	LS-SHOU, China
Escherichia coli ATCC8739	TSB	Biobw, China
Escherichia coli ATCC25922	LB	ATCC, United States
Escherichia coli K12	TSB	IIM, China
Enterobacter sakazakii CMCC45401	TSB	Biobw, China
Klebsiella pneumoniae 8-2-10-8	EE	LS-SHOU, China
Klebsiella pneumoniae 8-2-10-8	EE	LS-SHOU, China
Pseudomonas aeruginosa ATCC9027	TSB	Biobw, China
Pseudomonas aeruginosa ATCC27853	TSB	Biobw, China
Salmonella enterica subsp. *enterica* (*ex* Kauffmann and Edwards) Le Minor and Popoff serovar Choleraesuis ATCC13312	TSB	ATCC, United States
Salmonella paratyphi-A CMCC50093	TSB	GCCC, China
Salmonellaenterica subsp. *enterica* (*ex* Kauffmann and Edwards) Le Minor and Popoff serovar Vellore ATCC15611	TSB	ATCC, United States
Salmonella E1-1	LB	LS-SHOU, China
Shigella dysenteriae CMCC 51252	TSB	GCCC, China
Shigella flexneri CMCC51572	TSB	GCCC, China
Shigella flexneri ATCC12022	TSB	ATCC, United States
Shigella flexneri CMCC51574	TSB	GCCC, China
Shigella sonnei ATCC25931	TSB	ATCC, United States
Shigella sonnet CMCC51592	TSB	GCCC, China
Staphylococcus aureus ATCC 25923	TSB	ATCC, United States
Staphylococcus aureus ATCC 8095	TSB	ATCC, United States
Staphylococcus aureus ATCC29213	TSB	ATCC, United States
Staphylococcus aureus ATCC6538	TSB	ATCC, United States
Staphylococcus aureus D1-1	TSB	LS-SHOU, China
Vibrio alginolyticus ATCC17749	TSB	ATCC, United States
Vibrio alginolyticus ATCC33787	TSB	ATCC, United States
Vibrio cholerae GIM1.449	TSB	GCCC, China
Vibrio cholerae Q10-54	TSB	LS-SHOU, China
Vibrio fluvialis ATCC33809	Marine 2216	ATCC, United States
Vibrio harvey ATCC BAA-1117	Marine 2216	ATCC, United States
Vibrio harveyi ATCC33842	Marine 2216	ATCC, United States
Vibrio mimicus bio-56759	TSB	Biobw, China
Vibrio parahaemolyticus ATCC17802	TSB	ATCC, United States
Vibrio parahaemolyticus ATCC33847	TSB	ATCC, United States
Vibrio parahaemolyticus B3-13	TSB	LS-SHOU, China
Vibrio parahaemolyticus B4-10	TSB	LS-SHOU, China
Vibrio parahaemolyticus B5-29	TSB	LS-SHOU, China
Vibrio parahaemolyticus B9-35	TSB	LS-SHOU, China
Vibrio parahemolyticus A1-1	TSB	LS-SHOU, China
Vibrio vulnificus ATCC27562	TSB	Biobw, China

ATCC: American Type Culture Collection, United States; GCCC, Guangdong Culture Collection Center, Guangzhou, China; IIM, Institute of Industrial Microbiology, Shanghai, China; LS-SHOU, Laboratory stock, Shanghai Ocean University, Shanghai, China.

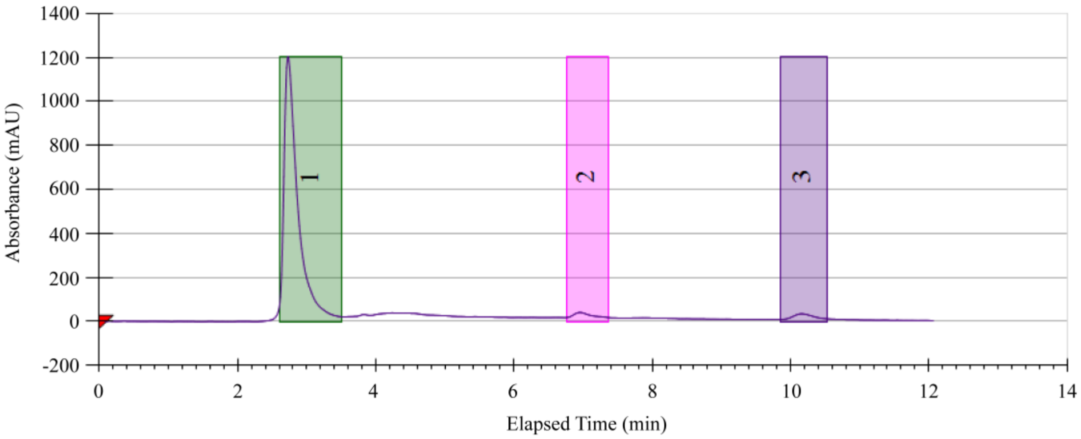

Figure S1. The Prep−HPLC diagram of purifying the methanol-phase crude extract from *P. kleiniana* Wight et Arn.
